# New data on known species of *Hirschmanniella* and *Pratylenchus* (Rhabditida, Pratylenchidae) from Iran and South Africa

**DOI:** 10.21307/jofnem-2019-041

**Published:** 2019-08-05

**Authors:** Ebrahim Shokoohi, Joaquín Abolafia, Phatu William Mashela, Nafiseh Divsalar

**Affiliations:** 1 Green Biotechnologies Research Centre of Excellence, University of Limpopo, Private Bag, X1106, Sovenga, 0727, South Africa; 2 Departamento de Biología Animal, Biología Vegetal y Ecología, Universidad de Jaén, Campus ‘Las Lagunillas’ s/n. 23071-Jaén, Spain; 3 Department of Plant Protection, Faculty of Agriculture, Shahid Bahonar University of Kerman, Kerman, Iran

**Keywords:** Iran, Morphometric, mtDNA, Phylogeny, Root-lesion nematode, rDNA, South Africa

## Abstract

*Hirschmanniella anchoryzae* from Iran and *Pratylenchus hippeastri* from South Africa were recovered during a survey of plant-parasitic nematodes belonging to the family Pratylenchidae. Both species were studied using morphological and molecular techniques. *Hirschmanniella anchoryzae* is identified based on the flattened head, short stylet (19–22 µm), excretory pore position (anterior to pharyngo-intestinal junction), spicule length (27–30 µm), and existence of an axial mucro at the tail end. Phylogenetic analysis using 28S rDNA showed monophyly of *Hirschmanniella* which Iranian *H. anchoryzae* placed close to *H. halophila* (EU620464; EU620465). This result was supported by the principal component analysis of *Hirschmanniella* species. SEM observation of the South African population of *P. hippeastri* showed the presence of two annuli in the lip region. Morphometric characters resembled those of specimens earlier reported from South Africa. Hierarchal cluster using morphometrical criteria showed that the Floridian (USA) and South African populations form a group. However, the principal component analysis showed variation within this species. The molecular study of *P. hippeastri* populations using 18S, ITS, 28S rDNA, and COI of mtDNA showed that all *P. hippeastri* cluster in one group and confirmed the identification of the species using both morphological and molecular techniques. In addition, the results indicated that South African populations group close to the USA populations. Illustrations of both species including light and scanning electron microscopy observations for *P. hippeastri* are provided.

The root-lesion nematodes belong to the family Pratylenchidae and cause severe damage on various crops and yield reduction (Perry and Moens, 2013). The genus *Hirschmanniella* has been established by Luc and Goodey (1964). To date, three known species of *Hirschmanniella*, namely, *H. anchoryzae* (Ebsary and Anderson, 1982), *H. gracilis* (De Man, 1880; Luc and Goodey, 1964), and *H. oryzae* (Van Breda de Haan, 1902; Luc and Goodey, 1964), and with two unknown *Hirschmanniella* have been reported from Iran (Majd Taheri et al., 2013). Those species have been studied by morphological characters except for two unknowns which have been studied by morphological and molecular DNA barcoding using 28S rDNA (Majd Taheri et al., 2013). Root-lesion nematodes, *Pratylenchus* (Filipjev, 1936), are after root-knot and cyst nematodes listed as the third economically most important genus that adversely affects crop production worldwide (Castillo and Vovlas, 2007; Jones et al., 2013). *Pratylenchus hippeastri*, the amaryllis lesion nematode, was first described by Inserra et al. (2007) from roots of *Hippeastrum* sp. in Florida (USA). Inserra et al. (2007) distinguished the species due to individuals having slender bodies, flat, plain, and smooth head regions with two lip annuli (some specimens with an incomplete third annulus) of which the second lip annulus is thicker than the first, ellipsoidal stylet knobs, rectangular and empty spermathecae with large round cavities, and conoid tails with bluntly pointed termini, usually with ventral constrictions or subhemispherical and smoothened. Three years later, the male of this species was described by De Luca et al. (2010) from bromeliads in Florida. Posteriorly, Gu et al. (2014) and Wang et al. (2016) reported the species from the rhizosphere of apple in Japan and China, respectively.

The main objectives of the present study were to (i) to identify the populations of *Hischmanniella* and *P. hippeastri* using morphology, morphometrics, and molecular DNA barcoding; (ii) to study of morphological variations among different *P. hippeastri* populations, and (iii) to determine the phylogenetic position of *H*. *anchoryzae* from Iran using 28S rDNA and *P. hippeastri* from South Africa using rDNA and mtDNA genes.

## Materials and methods

### Nematode materials

In 2015, *Hirschmanniella* specimens were collected from the rhizosphere of *Mentha aquatica* in Royan (Mazandaran Province, Iran) and *Pratylenchus* specimens were collected from rhizosphere soil samples of Cape Willow trees (*Salix mucronata*) growing on the banks of the Mooiriver in Potchefstroom (North-West Province, South Africa) and extracted from soil using the Whitehead tray method (Whitehead and Hemmings, 1965). Nematodes were fixed with a hot 4% formaldehyde solution and transferred to anhydrous glycerin (De Grisse, 1969). Measurements were done using an Olympus CH-2 and Omax light microscope (Nematology Laboratory; University of Limpopo) equipped with an ocular micro- and/or a curvimeter and drawing tube.

### Scanning electron microscopy (SEM)

Specimens preserved in glycerine were selected for observation under SEM according to Abolafia (2015). The nematodes were hydrated in distilled water, dehydrated in a graded ethanol-acetone series, critical point dried, coated with gold, and observed with a Zeiss Merlin microscope (5 kV) (Zeiss, Oberkochen, Germany).

### Statistical analysis

Principal component analysis and the correlation of morphometric data using the Pearson method was done by XLSTAT (Addinsoft, 2007). In total, 11 morphometric traits obtained from fixed nematodes including ‘de Man’s indices’ (*a*, *b*, *b′*, *c*, *c′*, and V), body length, stylet length, pharynx length, tail length, and position of the excretory pore were used for PCA analysis of *Hirschmanniella*. The species, namely, *H. halophila* (Germany: Sturhan and Hallmann, 2010), *H. loofi* (The Netherlands: Sher, 1968; Germany: Bert and Geraert, 2000), *H. kwazuna* (South Africa: Van den Berg et al., 2009), *H. pomponiensis* (USA: De Ley et al., 2007), *Hirschmanniella* sp. (Iran: Majd Taheri et al., 2013), *H. gracilis* (Iran: Jahanshahi Afshar et al., 2006), *H. oryzae* (India: Sher, 1968; Tiawan: Lin, 1970), *H. mucronata* (India, the Philippines and Thailand: Sher, 1968; Taiwan: Chen et al., 2006; Cambodia: Khun et al., 2015), *H. belli* (USA: Sher, 1968), *H. santarosae* (USA: De Ley et al., 2007), and *H. anchoryzae* (Canada: Ebsary and Anderson, 1982; Iran: Pourjam et al., 2000, present study) were studied. Regarding *Pratylenchus* species, hierarchical clustering analysis was done using morphometric data and the Rstudio, pvclust package (Suzuki and Shimodaira, 2015). To perform this analysis, 63 specimens of *P. hippeastri* were used. The averaged populations used for comparative purposes, hierarchical clustering as well as their morphometric data are available in the databases from the USA, two populations from Florida; for 32 specimens, respectively (Inserra et al., 2007; De Luca et al., 2012), 16 specimens from Japan (Gu et al., 2014), 10 specimens from China (Wang et al., 2016), and 5 specimens from South Africa as presented in the current study. In total, 14 morphometric traits obtained from fixed nematodes were used for identification purposes and hierarchical clustering analysis: ‘de Man’s indices’ (*a*, *b*, *b′*, *c*, *c′*, and V), body length, pharynx overlapping (distance from pharyngeal-intestinal junction to the end of overlapping), stylet length, DGO, MB (middle of metacorpus to anterior end), post vulva sac length, tail length, and position of the phasmid (Castillo and Vovlas, 2007). Data on the morphometric measurements of the populations were analyzed using the bootstrap method. The same morphometric characters except *b'* were used for PCA analysis of *P. hippeastri*.

### DNA extraction, PCR, and phylogenetic analysis

DNA extraction was done using the Chelex method (Straube and Juen, 2013). Five specimens of each species were hand-picked with a fine tip needle and transferred to a 1.5 ml Eppendorf tube containing 20 µl double distilled water. The nematodes in the tube were crushed with the tip of a fine needle and vortexed. In total, 30 µL of 5% Chelex® 50 and 2 µL of proteinase K were added to each of the microcentrifuge tubes that contained the crushed nematodes and mixed. These separate microcentrifuge tubes with the nematode lysate were incubated at 56°C for 2 hr, and then incubated at 95°C for 10 min to deactivate the proteinase K and finally spin for 2 min at 16,000 rpm (Shokoohi et al., 2018). The supernatant was then extracted from each of the tubes and stored at −20°C. Following this step, the forward and reverse primers, SSU F04 (5′-GCTTGTCTCAAAGATTAAGCC–3′) and SSU R26 (5′-CATTCTTGGCAAATGCTTTCG–3′); 18s (5′-TTGATTACGTCCCTGCCCTTT–3′) and 26s (5′-TTTCACTCGCCGTTACTAAGG–3′); D2A (5′-ACAAGTACCGTGAGGGAAAGTTG–3′), D3B (5′-TCGGAAGGAACCAGCTACTA–3′) and JB3 (5′-TTT TTT GGG CAT CCT GAG GTT TAT–3′), JB4.5 (5′-TAA AGA AAG AAC ATA ATG AAA ATG–3′) (Vrain et al., 1992; Blaxter et al., 1998; Subbotin et al., 2006; Derycke et al., 2010) were used in the PCR reactions for partial amplification of the 18S rDNA, ITS rDNA, 28S rDNA, and COI of mtDNA region. PCR was conducted with 8 µl of the DNA template, 12.5 µl of 2X PCR Master Mix Red (Promega, USA) for the South African specimens and (Pishgam, Iran) for the Iranian specimens), 1 µl of each primer (10 pmol µl^−1^), and ddH_2_O for a final volume of 30 µl. The amplification was processed using an Eppendorf master cycler gradient (Eppendorf, Hamburg, Germany), with the following program: initial denaturation for 3 min at 94°C, 37 cycles of denaturation for 45 sec at 94°C; 54°C, 55°C, 56°C, and 52°C annealing temperatures for 18S, ITS, 28S rDNA, and COI of mtDNA, respectively; extension for 45 sec to 1 min at 72°C, and finally an extension step of 6 min at 72°C followed by a temperature on hold at 4°C. Regarding *Hirschmanniella* only 28S rDNA and COI of mtDNA have been used for DNA amplification. After DNA amplification, 4 µl of product from each tube was loaded on a 1% agarose gel in TBE buffer (40 mM Tris, 40 mM boric acid, and 1 mM EDTA) for evaluation of the DNA bands. The bands were stained with RedGel (ethidium bromide for the Iranian specimens) and visualized and photographed on a UV transilluminator. The amplicons of each gene were stored at –20°C. Finally, the PCR products were purified for sequencing by Inqaba Biotech (South Africa) and Pishgam (Iran) for the Iranian specimens. Available sequences for other *Hirschmanniella* and *Pratylenchus* spp. were obtained from NCBI GenBank for comparison (Table 1). Also, as outgroups, *Pratylenchus vlunus* (EU130885) for *Hirschmanniella* based on the study of Khun et al. (2015), and *Zygotylenchus guevarai* (Tobar Jiménez, 1963; Braun and Loof, 1966) (AF442189; FJ717817; JQ917439) based on the study of Shokoohi (2013) were obtained for comparison of 18S, ITS, and 28S rDNA. *Rotylenchus macrosoma* (Dasgupta et al., 1968) (KY992847) was used as the outgroup for the COI of mtDNA analyses based on the study of Van Megen et al. (2009). The ribosomal and mitochondrial DNA sequences were analyzed and edited with BioEdit (Hall, 1999) and aligned using CLUSTAL W (Thompson et al., 1994). The length of the alignments was 1,794, 1,322, 838, and 445 bps for 18S, ITS, 28S rDNA, and COI of mtDNA, respectively, while the length of 28S rDNA alignment for *Hirschmanniella* species was 831 bp. Phylogenetic trees were generated using the Bayesian inference method as implemented in the program Mr Bayes 3.1.2 (Ronquist and Huelsenbeck, 2003). The GTR+I+G model was selected using jModeltest 2.1.10 (Guindon and Gascuel, 2003; Darriba et al., 2012). Then, the selected model was initiated with a random starting tree and ran with the Markov chain Monte Carlo (MCMC) for 10^6^ generations. The partial rDNA and COI of mtDNA sequences of *P. hippeastri* and 28S rDNA and COI of mtDNA of *H. anchoryzae* were deposited in GenBank and their accession numbers are shown in Table 2.

**Table 1.** List of the species used for phylogenetic analysis based on rDNA and mtDNA available in the GenBank for *Pratylenchus* and *Hirschmanniella*.

*Pratylenchus*								*Hirschmanniella*	
18S rDNA		ITS rDNA		28S rDNA		COI mtDNA		28S rDNA	
Species	Accession number/locality	Species	Accession number/locality	Species	Accession number/locality	Species	Accession number/locality	Species	Accession number/locality
*P. speijeri*	KM245059/China	*P. parafloridensis*	GQ988378/USA	*P. hippeastri*	FN554882/USA	*P. coffeae*	KU198943/Japan	*H. oryzae*	JX291141/Myanmar
*P. speijeri*	KF974690/China	*P. parafloridensis*	GQ988377/USA	*P. hippeastri*	FM994115/USA	*P. coffeae*	KU198942/Japan	*H. oryzae*	JX291142/Myanmar
*P. coffeae*	AB905286/Japan	*P. floridensis*	GQ988375/USA	*P. hippeastri*	FN554881/USA	*P. coffeae*	KY424075/China	*Hirschmanniella* sp.	DQ328686/Vietnam
*P. coffeae*	KM245066/China	*P. floridensis*	GQ988376/USA	*P. hippeastri*	FN554879/USA	*P. coffeae*	KY424074/China	*H. oryzae*	KF201169/the Philippines
*P. coffeae*	KY424134/China	*P. hippeastri*	FJ712933/USA	*P. hippeastri*	FM994114/USA	*P. speijeri*	KY424088/China	*H. oryzae*	KF201165/the Philippines
*P. speijeri*	KF974688/ China	*P. hippeastri*	FJ712935/USA	*P. hippeastri*	KP161611/China	*P. speijeri*	KY424087/China	*H. oryzae*	KF201161/the Philippines
*P. coffeae*	KY424139/China	*P. hippeastri*	FJ712934/USA	*P. hippeastri*	KC796704/Japan	*P. loosi*	KY424086/Japan	*H. belli*	EF029860/USA
*P. coffeae*	KY424140/China	*P. hippeastri*	FJ712936/USA	*P. hippeastri*	KC796705/Japan	*P. loosi*	KY424085/China	*Hirschmanniella* sp.	EF029861/USA
*P. coffeae*	KY424142/China	*P. hippeastri*	KY424236/China	*P. hippeastri*	KC796706/Japan	*P. loosi*	KY424084/China	*Hirschmanniella* sp.	KP671713/Belgium
*P. speijeri*	KY424156/China	*P. hippeastri*	KR029085/China	*P. hippeastri*	KC796707/Japan	*P. loosi*	KX349422/China	*H. kwazuna*	South Africa
*P. speijeri*	KM245067/China	*P. hippeastri*	KY424237/China	*P. hippeastri*	KY424307/China	*P. hippeastri*	KY424099/China	*H. kwazuna*	South Africa
*P. coffeae*	KY424137/China	*P. hippeastri*	KC796698/Japan	*P. hippeastri*	KR029084/China	*P. hippeastri*	KY424098/China	*H. loofi*	EU620468/Belgium
*P. coffeae*	KY424143/China	*P. hippeastri*	KC796701/Japan	*P. hippeastri*	KY424306/China	*P. scribneri*	KY424092/China	*H. loofi*	EU620469/Belgium
*P. loosi*	AB905296/Japan	*P. hippeastri*	KC796702/Japan	*P. hippeastri*	KJ001720/China	*P. scribneri*	KX349425/China	*Hirschmanniella* sp.	JX261958/Iran
*P. loosi*	KY424153/China	*P. hippeastri*	KC796699/Japan	*P. hippeastri*	GU214112/USA	*P. scribneri*	KY424090/China	*H. mucronata*	KP179327/Cambodia
*P. loosi*	KY424154/China	*P. hippeastri*	KJ001718/Israel	*P. hippeastri*	KC796703/Japan	*P. scribneri*	KY424091/China	*H. mucronata*	KF201167/the Philippines
*P. loosi*	KY424155/China	*P. hippeastri*	FJ712932/USA	*P. parafloridensis*	GU214114/USA	*P. scribneri*	KY424089/China	*H. mucronata*	KP179333/Cambodia
*P. loosi*	AB905297/Japan	*P. hippeastri*	KC796700/Japan	*P. parafloridensis*	AF170438/USA	*P. vulnus*	KY828317/Belgium	*H. halophila*	EU620464/Germany
*P. agilis*	EU130794/USA	*P. hippeastri*	FN554883/USA	*P. parafloridensis*	GU214115/USA	*P. vulnus*	KY828312/Belgium	*H. halophila*	EU620465/Germany
*P. agilis*	EU130793/USA	*P. hippeastri*	FN554887/USA	*P. floridensis*	GU214116/USA	*P. vulnus*	KY424096/China	*H. pomponiensis*	DQ077795/USA
*P. scribneri*	EU130812/USA	*P. hippeastri*	FN554884/USA	*P. floridensis*	AF170437/USA	*P. vulnus*	KY424094/China	*H. santarosae*	EF029859/USA
*P. scribneri*	EU130811/USA	*P. hippeastri*	FN554888/USA	*P. floridensis*	GU214117/USA	*P. oleae*	KJ510866/Spain	*P. vulnus*	EU130885/USA
*P. scribneri*	KY424158/China	*P. jaehni*	FJ712941/Brazil	*P. araucensis*	FJ463261/Colombia	*Rotylenchulus macrosoma*	KY992847/Greece		
*P. scribneri*	KY424159/China	*P. jaehni*	FJ712940/Brazil	*P. araucensis*	FJ463258/Colombia				
*P. scribneri*	EU669927/the Netherlands	*P. loosi*	FJ712946/Brazil	*P. araucensis*	FJ463260/Colombia				
*P. scribneri*	KY424162/China	*P. loosi*	FJ712942/Brazil	*P. coffeae*	AF170427/USA				
*P. scribneri*	KY424161/China	*P. pseudocoffeae*	KT971367/Costa Rica	*P. coffeae*	AF170426/USA				
*P. hippeastri*	KY424166/China	*P. pseudocoffeae*	KT175523/South Korea	*P. pseudocoffeae*	KT175531/South Korea				
*P. hippeastri*	KJ001716/Israel	*P. pseudocoffeae*	LC030339/Japan	*P. pseudocoffeae*	KT175532/South Korea				
*P. scribneri*	EU669958/the Netherlands	*P. pseudocoffeae*	LC030338/Japan	*P. pseudocoffeae*	KT971360/Costa Rica				
*P. araucensis*	FJ154950/Colombia	*P. scribneri*	KY424228/China	*P. pseudocoffeae*	KT175533/South Korea				
*P. japonicus*	KF385443/Japan	*P. scribneri*	KY424230/China	*P. scribneri*	KY424300/China				
*P. parazeae*	KY424184/China	*P. agilis*	FJ712891/USA	*P. agilis*	EU130841/USA				
*P. pratensis*	KC875387/the Netherlands	*P. agilis*	JQ039330/China	*P. scribneri*	JX047002/China				
*P. bolivianus*	KC875390/the Netherlands	*P. alleni*	JX081545/Canada	*P. scribneri*	EU130865/USA				
*Zygotylenchus guevarae*	AF442189/Belgium	*P. gutierrezi*	FJ712929/Guatemala	*P. scribneri*	KX842632/USA				
		*P. gutierrezi*	FJ712930/Guatemala	*P. alleni*	MF155653/Canada				
		*P. gutierrezi*	FJ712931/Guatemala	*P. speijeri*	KF974713/China				
		*P. gutierrezi*	FR692277/Portugal	*P. speijeri*	KF974715/China				
		*Zygotylenchus guevarai*	FJ717817/Spain	*P. speijeri*	KY424295/China				
				*P. speijeri*	KF974716/China				
				*P. speijeri*	KF974703/China				
				*P. coffeae*	EU130846/Japan				
				*P. coffeae*	EU130850/Japan				
				*P. coffeae*	EU130845/Japan				
				*P. coffeae*	KC490925/China				
				*P. loosi*	KY424290/China				
				*P. loosi*	EF446995/Iran				
				*P. loosi*	KY424291/China				
				*P. loosi*	KY424293/China				
				*P. loosi*	JN091970/Japan				
				*P. penetrans*	JX046999/China				
				*P. penetrans*	JX046998/China				
				*P. dunensis*	AJ890462/the Netherlands				
				*P. dunensis*	AJ890460/the Netherlands				
				*P. brachyurus*	KF712474/China				
				*P. brachyurus*	KF712472/China				
				*P. vulnus*	HM469437/China				
				*P. vulnus*	KF430799/Japan				
				*P. crenatus*	KX683378/the Netherlands				
				*P. crenatus*	EU130853/UK				
				*P. bhattii*	JN244270/China				
				*P. bhattii*	JN244269/China				
				*P. parazeae*	KP903445/China				
				*P. parazeae*	KP903443/China				
				*P. zeae*	KT033000/Kenya				
				*P. zeae*	KT032999/Kenya				
				*P. bolivianus*	KU198956/Bolivia				
				*P. bolivianus*	KU198955/Bolivia				
				*P. neglectus*	HM469438/China				
				*P. neglectus*	MG205581/China				
				*P. brzeskii*	AM231928/France				
				*P. brzeskii*	AM231927/France				
				*P. thornei*	KX258736/Iran				
				*P. thornei*	KX258737/Iran				
				*P. thornei*	EU130881/Moldova				
				*Zygotylenchus guevarai*	JQ917439/Iran				

**Table 2.** Nematode species and GenBank accession numbers used for the present study.

Species	Gene	GenBank accession number	Origin	Sample codes
*P. hippeastri*	18S rDNA	MH324470	Potchefstroom, South Africa	ESW 1
*P. hippeastri*	ITS rDNA	MH324471	Potchefstroom, South Africa	ESW 2
*P. hippeastri*	28S rDNA	MH324472	Potchefstroom, South Africa	ESW 3
*P. hippeastri*	28S rDNA	MH324473	Potchefstroom, South Africa	ESW 4
*P. hippeastri*	COI of mtDNA	MH324474	Potchefstroom, South Africa	ESW 5
*H. anchoryzae*	28S rDNA	MK571451	Royan, Iran	IR Royan
*H*. *anchoryzae*	COI of mtDNA	MK583962	Royan, Iran	IR Royan

## Results

### *Hirschmanniella anchoryzae* (Ebsary and Anderson, 1982)

(Fig. 1; Table 3).

**Table 3.** Morphometrics of *H. anchoryzae* from Iran and *P. hippeastri* from South Africa. All measurements are in μm and in the form: mean ± s.d. (range).

Species	*H. anchoryzae*		*P. hippeastri*
Locality	Royan		Potchefstroom
Province	Mazandaran Province		North-West Province
Country	Iran		South Africa
Habitat	*Mentha aquatica*		Willow tree
n	4 ♀♀	2 ♂♂	7 ♀♀
L	1,796 ± 71 (1,740–1,895)	1,273, 1,625	522.6 ± 69.5 (424–614)
*a*	65.0 ± 2.0 (63.6–68.0)	62.1, 62.5	28.5 ± 3.1 (18.6–31.8)
*b*	10.3 ± 1.4 (9.0–11.6)	6.6, 8.3	3.8 ± 0.7 (2.7–4.9)
*c*	18.6 ± 2.3 (16.4–21.9)	15.7, 18.8	18.7 ± 3.1 (11.7–23.6)
*c′*	5.5 ± 1.6 (4.2–7.2)	4.2, 5.4	2.2 ± 0.2 (2.0–2.3)
V	55.4 ± 1.9 (53–57)	–	77.1 ± 3.5 (73–82)
Lip region height	4.1 ± 0.2 (4–5)	3, 4	2.6 ± 0.7 (1.9–3.7)
Lip region diameter	10.3 ± 0.5 (10–11)	9, 11	8.5 ± 1.7 (7–11)
Stylet length	20.2 ± 1 (19–21)	18, 19	15.4 ± 1.6 (13–18)
Stylet conus length	9.8 ± 0.7 (9–11)	9, ?	7.6 ± 0.7 (6.4–8.2)
Stylet shaft length	8.4 ± 0.5 (8–9)	8, ?	4.9 ± 1.2 (4.2–5.8)
Stylet knob height	1.7 ± 0.2 (1.5–1.8)	1.5, ?	2.2 ± 0.4 (1.7–2.5)
Stylet knob width	3.6 ± 0.3 (3.3–3.8)	3.4, ?	3.4 ± 0.7 (2.7–4.1)
DGO from stylet base	3.2 ± 0.4 (3–4)	3.3, 3.7	2.7 ± 0.9 (1.7–3.6)
Anterior end to centre of median bulb	87 ± 3 (84–91)	77, 93	53.8 ± 3.7 (50–60)
End of pharyngeal glands	393 ± 13 (380–410)	223, 441	132.9 ± 15.9 (116–149)
Median bulb length	12.7 ± 0.2 (12–13)	13	13.8 ± 2.7 (11–16)
Median bulb width	16.6 ± 0.5 (16–17)	18	9.1 ± 1.8 (7–10)
Excretory pore – anterior end	150 ± 2 (148–152)	119, 143	95.7 ± 15.1 (80–112)
Maximum body diameter	28 ± 2 (26–29)	21, 26	20.9 ± 3.4 (17–27)
Anal body diameter	18 ± 4 (13–20)	15, 20	12.9 ± 1.0 (12–14)
Anterior genital tract length	?	–	160.5 ± 36.5 (102–210)
Tail length	101 ± 4 (96–106)	81, 87	31.7 ± 6.4 (26–43)
Number of tail annuli	73 ± 9 (63–80)	?	21.3 ± 2.8 (18–23)
Vulva to anus distance	–	–	77.5 ± 7.7 (72–83)
Post-vulval uterine sac length	–	–	20.6 ± 3.9 (16–26)
Lateral field width	7.5	7	5.5 ± 0.7 (5–6)
Phasmid-anus distance	57.8 ± 7.6 (46–64)	49, 61	17.2 ± 4.1 (15-22)
Spicules	–	27, 30	–
Gubernaculum	–	8.6-8.7	–

**Figure 1:**
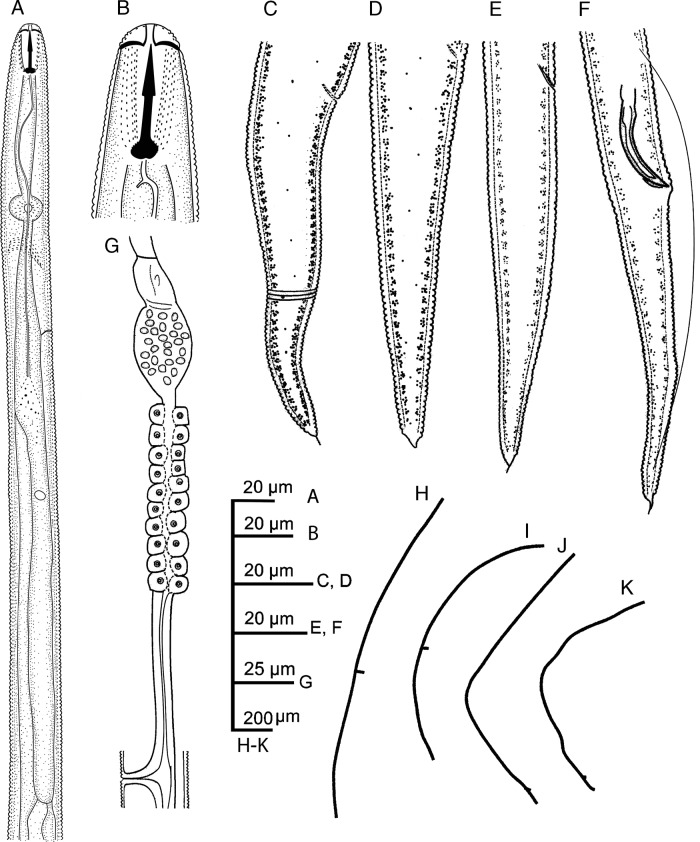
Line drawings of *Hirschmanniella anchoryzae*. (A) anterior portion of the female; (B) cephalic region of the female; (C–E) female posterior end; (F) male posterior end; (G) female reproductive system; (H, I) status of females after relaxation; (J, K) status of male after relaxation.

#### Females

The description of female body of *Hirschmanniella anchoryzae* is as follows: body length is from 1,740 to 1,895 µm, slightly curved to ‘C’ shaped after fixation, some specimens slightly straight; cuticle is finely annulated with 1.2 to 1.5 µm wide at midbody; maximum body diameter is of 26 to 29 µm; cephalic region is continuous with the body; lip region is flat, with a slight depression, bearing four to five annuli; lateral field is with four lines, 8 to 10 µm width, occupying about 31 to 34% of midbody diameter, aereolated along the body especially in the tail region; stylet length is from 19 to 21 µm, basal knobs usually rounded, and stylet conus 46 to 50% of the total length of the stylet; dorsal pharyngeal gland opening (DGO) is at 3 to 4 µm posterior to stylet base; median bulb is spherical to oval with 7 to 11×9 to 10 µm length and width, respectively; nerve ring is located just after isthmus, at 27 to 28% of the neck (from head to end of pharyngeal gland overlapping); excretory pores are at 1 to 8 µm anterior to the pharyngeal-intestinal junction, at 35 to 37% of the neck; hemizonid is 5 to 8 µm anterior to excretory pore; pharyngeal glands are overlapped with 8 to 10 times than the corresponding body diameter; pharynx is 380 to 410 µm long, about 22% of the body length; in reproductive system didelphic amphidelphic, ovary is not reaching the pharyngeal glands; oocytes are in one or two rows; vulva occupies 35 to 40% of the corresponding body diameter; spermatheca is visible, oval shape, with sperm; vulva is with a transverse slit with not protruded lips, V = 53 to 57; tail is 96 to 106 µm long, conical, elongated with 63 to 80 ventral annuli and axial mucro, in some specimens the appendage visible as a notch; and phasmid is located at about middle of the tail, 57 to 61% of the tail length.

#### Male

The structure of male body is similar to the female body with hypoptygma. Reproductive system is monorchid. Spicules tylenchoidis of 30 to 35 µm length, paired, separate in ventral view, smooth, and ventrally arcuate in lateral view; rounded manubrium; calamus very short, lamina thin, ventral curved end. In lateral view, the gubernaculum is bent, 5 to 8 µm length, 18 to 26% of spicule length. Bursa is leptoderan. Phasmid is 60 to 70% of the tail length. Tail is conical, elongated, 81 to 87 µm with axial mucro at tail tip.

#### Remarks

Four females from Royan (Mazandaran Province, Iran) in a good state of preservation were studied. Iranian population of *Hirschmanniella* is similar to *H. anchoryzae* based on the original description and the identification key (Ebsary and Anderson, 1982). In comparison with the *H. anchoryzae* reported previously from Iran (Pourjam et al., 2000), they differ in female body length (1,740–1,895 vs 1,580–1,680 µm) and spicule length (27–30 µm vs 39–40 µm). This population differentiate from *H. oryzae* (Luc and Goodey, 1964) in having longer spicule (27–30 µm vs 18–26 µm) and shorter gubernaculum (5–8 µm vs 9–14 µm) (Khan and Bala, 2003). This population also resembles *H. gracilis* (De Man, 1880; Luc and Goodey, 1964). However, the two differed in the upper range of the body length (1.9 and 1.6 mm for female and male, respectively, vs 2.2 and 2.0 mm for female and male, respectively), stylet length (19–21 µm vs 20–24 µm), and gubernaculum length (5–8 µm vs 9–15 µm) (Loof, 1991).

#### PCA analysis of H. anchoryzae

**Figure 2:**
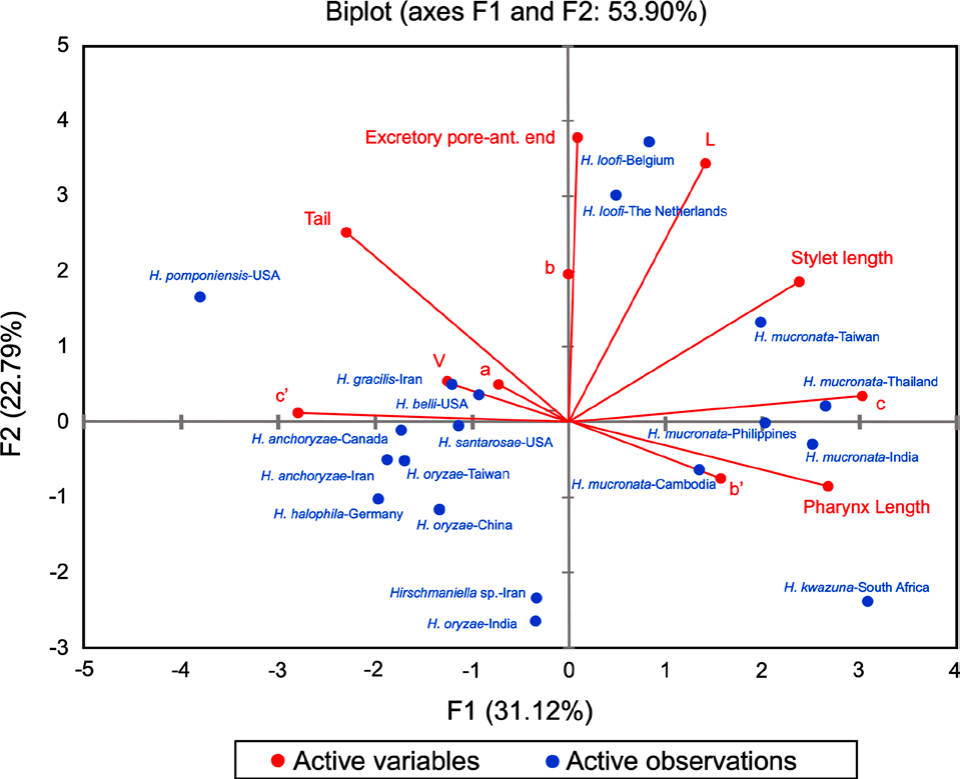
PCA analysis of the different population of *H. anchoryzae*.

The principal component analysis was performed to study the variation within the populations of *H. anchoryzae* (Fig. 2). An accumulated variability of 53.90% was detected in the female by the F1 (31.12%) and F2 (22.79%). All characters exhibited positive correlations among the populations and were responsible for the variability of the F1, except for *a*, *b*, *c′* and tail length (Fig. 2). Some characters such as *c*, *c′*, pharynx length, stylet length, and tail length with 22.1, 18.8, 17.1, 13.5, and 12.6% showed the most contribution to the variability and had a high correlation with the F1, while excretory pore to anterior end and body length with 34.3 and 28.2% showed a high correlation with the F2. The result indicated that two population of *H. anchoryzae* from Iran and Canada place close each other. Two populations of *H. anchoryzae* place close with the *H. halophila* from Germany.

#### DNA characterization

The genes 28S rDNA and COI of mtDNA for *H. anchoryzae* yielded 687 and 385 bp, respectively. Nblast of the 28S rDNA showed 93% identity with a Dutch population (acc. nr: EU620464; EU620465) of *H. halophila* (Sturhan and Hallmann, 2010) with 35 nucleotides differences. Compare with a population of *H. santarosae*, De Ley et al. (2007) showed 90% identity with 59 nucleotide differences. In comparison with *H. pomponienis*, Abdel-Rahman and Maggenti (1987) showed 89% identity with 70 nucleotide differences. Regarding COI of mtDNA, Nblast showed 95% identity with an unidentified population (acc. nr: KX349428) of *Hirschmanniella* from China showing 18 nucleotide differences. In comparison with *H. mucronata* (KY424110), our sequence showed 84% identity with 61 nucleotide differences, whereas another population of *H. mucronata* (KR819278) from China there was 71% identity with 89 nucleotide differences. Genetic pairwise distance (Table 4) indicated the lowest (0.073) and the highest range (0.166) among *H. anchoryzae* (MK571451), obtained from *H. halophila* (EU620464, EU620465) and *H. mucronata* (KP179327, KP179333, KF201167), respectively. The lowest range (0.000) was observed between an unidentified *Hirschmanniella* (DQ328686) from Vietnam and *H. oryzae* from Myanmar and the Philippines.

**Table 4.** Genetic pairwise distance estimation of 28S rDNA of *Hirschmanniella* species using Mega 7.

	Species	Locality	1	2	3	4	5	6	7	8	9	10	11	12	13
1	*H. anchoryzae*	Iran		0.035	0.049	0.051	0.032	0.048	0.055	0.052	0.053	0.056	0.024	0.058	0.053
2	*H. pomponiensis*	USA	0.109		0.040	0.039	0.007	0.038	0.045	0.044	0.044	0.047	0.024	0.047	0.041
3	*Hirschmanneilla* sp.	Iran	0.147	0.126		0.031	0.038	0.027	0.030	0.030	0.016	0.014	0.046	0.022	0.030
4	*Hirschmanneilla* sp.	USA	0.146	0.122	0.102		0.035	0.012	0.014	0.014	0.031	0.030	0.040	0.031	0.004
5	*H. santarosae*	USA	0.101	0.019	0.123	0.110		0.033	0.039	0.038	0.039	0.042	0.022	0.042	0.036
6	*H. belli*	USA	0.137	0.116	0.088	0.036	0.103		0.007	0.006	0.026	0.026	0.038	0.031	0.011
7	*H. oryzae*	Myanmar and the Philippines	0.152	0.131	0.097	0.043	0.119	0.016		0.000	0.029	0.028	0.042	0.031	0.012
8	*Hirschmanneilla* sp.	Vietnam	0.146	0.129	0.098	0.044	0.117	0.015	0.000		0.030	0.029	0.044	0.031	0.012
9	*H. kwazuna*	South Africa	0.149	0.131	0.050	0.097	0.119	0.082	0.090	0.092		0.010	0.039	0.018	0.028
10	*H. loofi*	Belgium	0.157	0.139	0.044	0.097	0.128	0.083	0.089	0.092	0.026		0.041	0.017	0.028
11	*H. halophila*	Germany	0.073	0.075	0.140	0.121	0.069	0.113	0.124	0.128	0.118	0.124		0.043	0.040
12	*H. mucronata*	Belgium	0.166	0.145	0.074	0.103	0.132	0.100	0.100	0.100	0.058	0.055	0.132		0.031
13	*Hirschmanneilla* sp.	Belgium	0.149	0.124	0.098	0.009	0.111	0.030	0.036	0.036	0.089	0.089	0.120	0.101	

Notes: Accession numbers: 1 = MK571451; 2 = DQ077795; 3 = JX261958; 4 = EF029861; 5 = EF029859; 6 = EF029860; 7 = JX291141, JX291142, KF201161, KF201165, KF201169; 8 = DQ328686; 9 = EU620466, EU620467; 10 = EU620468, EU620469; 11 = EU620464, EU620465; 12 = KP179327, KP179333, KF201167; 13 = KP671713.

#### Phylogenetic analysis

**Figure 3:**
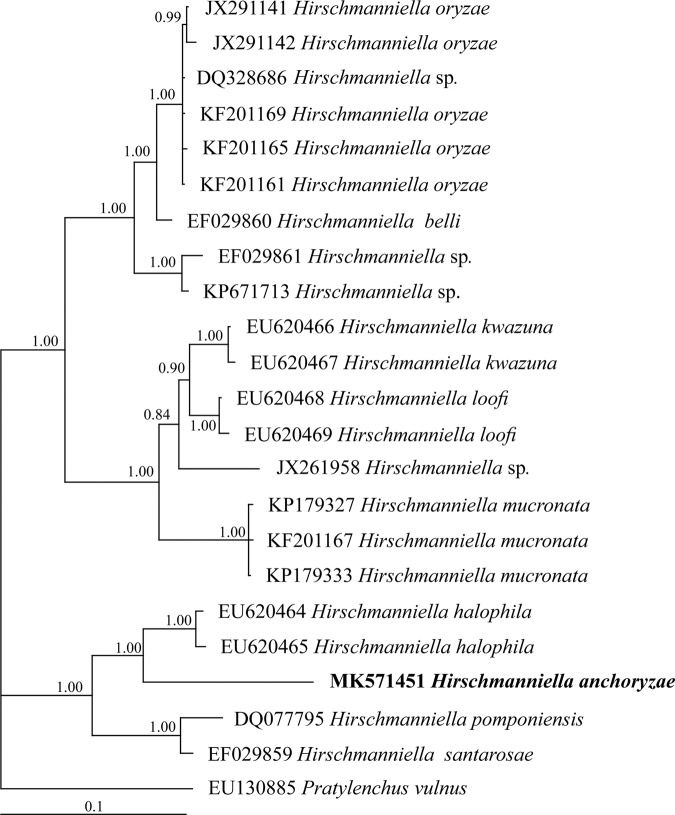
The Bayesian inference tree of *Hirschmanniella anchoryzae* (Ebsary and Anderson, 1982) from Iran and other related species based on the sequences from 28S rDNA under GTR+I+G model (−*lnL* = 3,374.3581; AIC = 6,856.7162; freqA = 0.2269; freqC = 0.2193; freqG = 0.3068; freqT = 0.2471; R(a) [AC] = 0.6057; R(b) [AG] = 2.7984; R(c) [AT] = 0.9170; R(d) [CG] = 0.2612; R(e) [CT] = 3.5814; R(f) [GT] = 1; p-inv = 0.1960; shape = 0.5340).

The Bayesian inference tree of 28S rDNA of *Hirschmanniella* species (Fig. 3) grouped them into three clades including (i) *H. oryzae*, *H. belli* (Sher, 1968), and unidentified *Hirschamnniella* with 1.00 posterior probability; (ii) *H. loofi*, *H. kwazuna*, *H. mucronata*, and unidentified *Hirschamnniella* with 1.00 posterior probability, and (iii) *H*. *anchoryzae*, *H. pomponiensis*, *H. santarosae*, and *H. halophila* with 1.00 posterior probability.

### *Pratylenchus hippeastri* (Inserra et al., 2007)

**Figure 4:**
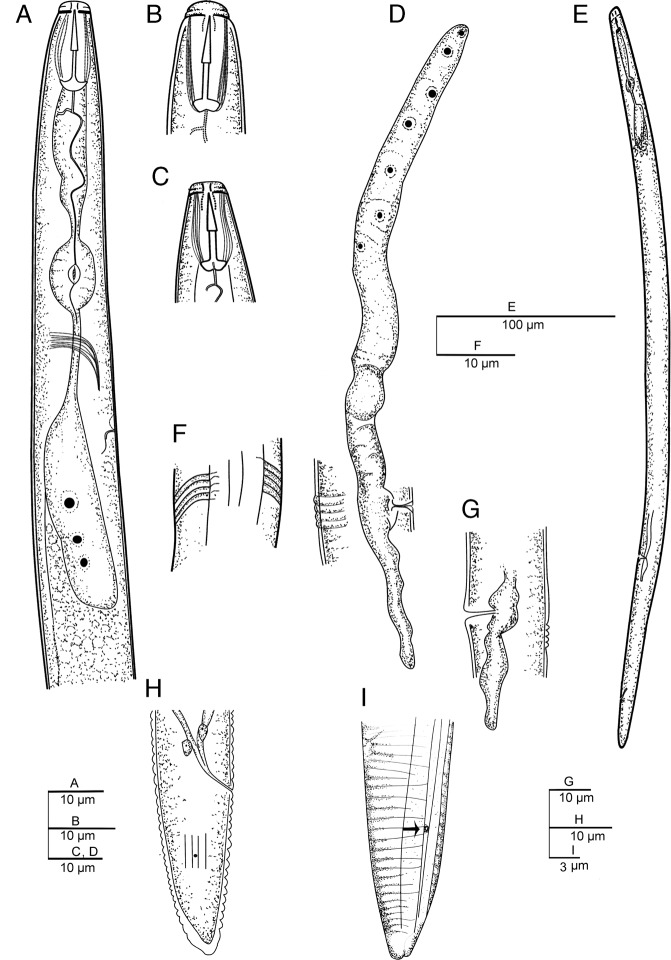
Line drawings of *Pratylenchus hippeastri*. (A) female anterior end; (B, C) stoma; (D) female reproductive system; (E) entire female; (F) lateral field; (G) post uterine sac; (H, I) female posterior end (arrow indicates phasmid).

**Figure 5:**
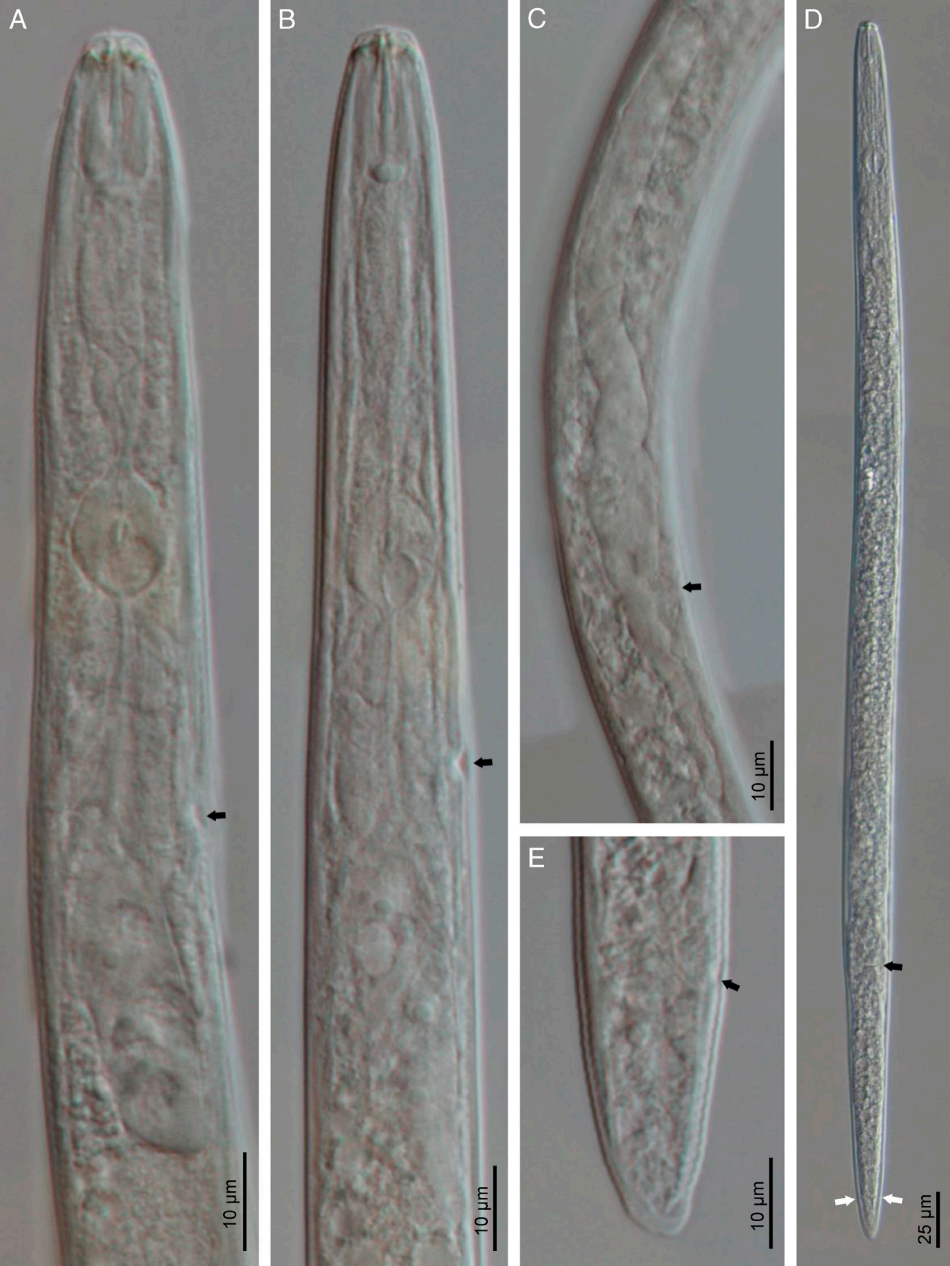
Light photomicrographs of *Pratylenchus hippeastri*. (A, B) anterior end (arrows indicate hemizonid); (C) reproductive system (arrow indicates vulva); (D) entire body (black arrow indicates vulva, white arrows indicate phasmids); (E) posterior end (arrow indicate anus).

**Figure 6:**
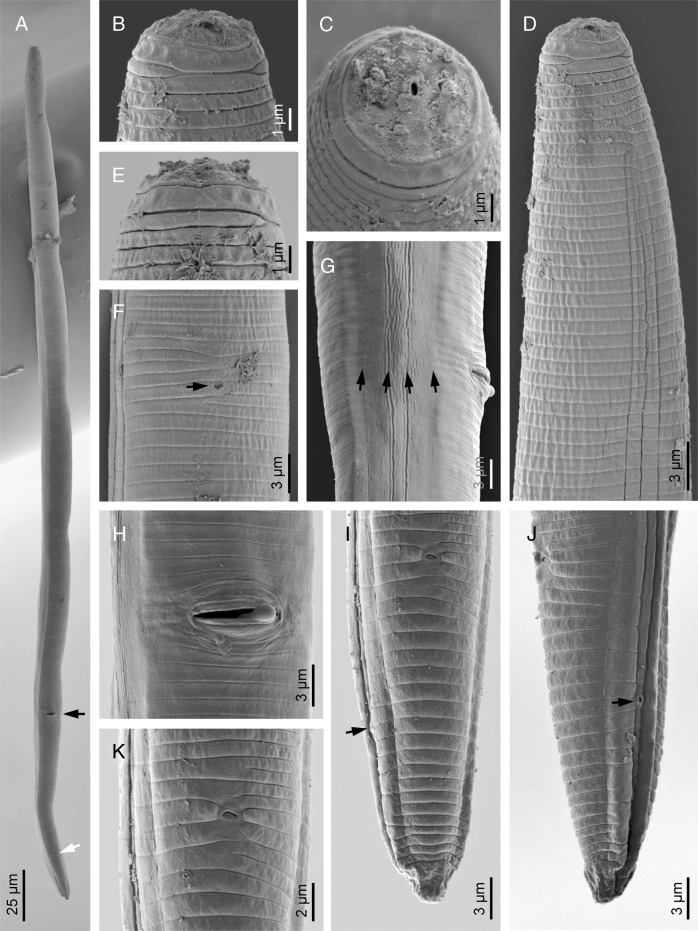
Scanning electron microscope photographs of *Pratylenchus hippeastri*. (A) entire body (black arrow indicates vulva); (B, C, E) lip region in lateral, frontal and ventral views, respectively); (D) female anterior region; (F) excretory pore (arrow); (G) lateral field (arrows indicate longitudinal incisures); (H) vulval region; (I, J) female posterior end in ventral and lateral views, respectively (arrow indicates phasmid); (K) anus.

(Figs. 4–6; Table 3).

#### Females

The description of female body of *Pratylenchus hippeastri:* body length is 423 to 614 µm, slightly curved after fixation, some specimens straight; cuticle is finely annulated with 0.9 to 1.2 µm wide at midbody; maximum body diameter is 17 to 27 µm; cephalic region is continuous with the body; lip region is round to flat, with a slight depression, bearing two annuli; lateral field with four lines, started with two lines 7.5 µm from anterior end, ending at tail terminus with three lines, occupies about 25 to 36% of midbody diameter and around 53% at the vulval region; stylet length is 13.3 to 18.0 µm, basal knobs usually rounded and flatted; dorsal esophageal gland opening (DGO) is at 1.7 to 3.6 µm posterior to stylet base; median bulb oval, nerve ring located just after isthmus, is at 48 to 66% of the neck; excretory pore is at 80 to 112 µm from the anterior body, at 60 to 70% of the neck; hemizonid one annuli is anterior to excretory pore; pharyngeal glands overlapped with intestine are about 32 to 43 µm; neck is 116 to 149 µm long, body length about 3.5 to 4.9 times than pharynx length; ovary is not reaching the pharyngeal glands; columnar cells of uterus are distinct and disposed in four rows; oocytes are in one row at growth zone (V = 75-82); spermatheca is visible, circular to oval shape, without sperm; post-vulval uterine sac is 11 to 26 µm long, vulva with a transverse slit and protruded lips; tail is conoid with bluntly pointed, usually with a ventral constriction at the middle or subhemispherical, slightly smooth terminus; tail is with slightly indented terminus observed in some specimens; tail is 26 to 43 µm long, about 2.0 to 2.5 times than anal body diameter; phasmid is located at about middle of the tail, 46 to 55% of the tail length; and the hyaline portion of tail terminus is 2.5 to 3.8 µm long.

#### Male

Not found.

#### Remarks

Seven females from Potchefstroom (South Africa) in a good state of preservation were studied. This species was previously reported from Florida, USA (Inserra et al., 2007; De Luca et al., 2012), Japan (Gu et al., 2014), and China (Wang et al., 2016). In the present study, this species has been studied from South Africa. The morphometric characters resemble those studied previously. However, compared to the specimens studied from Florida (Inserra et al., 2007), the specimens from Potchefstroom have shorter tail lengths (26–33 μm vs 32–42 μm) and shorter post uterine sacs (11–26 μm vs 21–45 μm). The lip region is reported to contain two annuli with the first being deeper than the second one (Inserra et al., 2007; De Luca et al., 2012; Wang et al., 2016). The results indicated the uncompleted third incisure in some specimens of *P. hippeastri*. SEM observation of the Potchefstroom individuals indicated that two lip annuli are present, in which the first one being deeper. Uncompleted lip annuli and tail variation forms were not observed in Potchefstroom specimens. According to the identification key presented by Inserra et al. (2007), *P. hippeastri* is similar to *P*. *scribneri* Steiner in Sherbakoff and Stanley’s (1943) study reported from Ohio (USA). These authors separated *P. hippeastri* from *P. scribneri* by a longer tail (36.6 vs 26.7 μm), slightly longer stylet (15.4 vs 14.7 μm), and shape of tail terminus, but often bluntly pointed and smooth terminus vs the consistently hemispherical and smooth tail terminus. The present results indicated that the stylet is overlapped in the two mentioned species (stylet length 13.3-15.4 µm in South African population in this study). Concerning the tail end morphology, none of the South African specimens studied had smooth ends. Hence, based on the results of this study, the tail morphology of *P. hippeastri* seems to be different from that of *P. scribneri* as shown in the study of Inserra et al. (2007). However, more specimens from different localities of South Africa are needed for the detailed description of the test populations.

#### Morphometric analysis on the P. hippeastri

The results based on two-tailed Pearson correlation within the five *P. hippeastri* populations studied showed that some morphometric data, obtained from 63 females (except for the South African specimens, the average based on the available data have been used), showed significant correlations with others. Some important morphometric data such as body length seemed to be considered to understand the correlation with each other. Results indicated that body length showed the highest correlation with the *b* (*r* = 0.786) and tail (*r* = 0.768), respectively (Table 5). On the other hand, body length had no significant correlation with some morphometric characters such as stylet length (*r* = 0.277). Despite the stylet length, body length showed the highest significant correlation (*r* = 0.931, P = 0.05) with MB (median bulb to anterior end as % of the pharynx). Tail length showed a highly significant correlation with the index *b* (*r* = 0.904).

**Table 5.** Correlation of morphometric data of *P. hippeastri* from South Africa.

Variables	L	*a*	*b*	*c*	*ć*	V	Stylet	DGO	Tail	PUS	Phasmid	MB	Pharynx
L	*1*	−0.236	0.786	0.336	−0.340	−0.453	0.277	−0.404	0.768	0.672	0.626	*0.931*	−0.841
*a*	−0.236	*1*	−0.516	0.589	−0.210	−0.285	−0.601	−0.166	−0.605	−0.846	0.001	−0.449	−0.205
*b*	0.786	−0.516	*1*	−0.187	0.198	−0.503	0.792	−0.287	*0.904*	0.806	0.797	0.709	−0.679
*c*	0.336	0.589	−0.187	*1*	*−0.882*	0.082	−0.488	−0.692	−0.344	−0.191	−0.134	0.140	−0.359
*ć*	−0.340	−0.210	0.198	*−0.882*	*1*	−0.427	0.471	0.607	0.275	−0.079	0.407	−0.280	0.103
V	−0.453	−0.285	−0.503	0.082	−0.427	*1*	−0.181	−0.141	−0.543	0.059	*−0.913*	−0.329	0.811
Stylet	0.277	−0.601	0.792	−0.488	0.471	−0.181	*1*	−0.257	0.588	0.679	0.531	0.192	−0.197
DGO	−0.404	−0.166	−0.287	−0.692	0.607	−0.141	−0.257	*1*	0.089	−0.268	−0.094	−0.120	0.347
Tail	0.768	−0.605	*0.904*	−0.344	0.275	−0.543	0.588	0.089	*1*	0.771	0.736	0.831	−0.614
PUS	0.672	−0.846	0.806	−0.191	−0.079	0.059	0.679	−0.268	0.771	*1*	0.296	0.738	−0.261
Phasmid	0.626	0.001	0.797	−0.134	0.407	*−0.913*	0.531	−0.094	0.736	0.296	*1*	0.472	−0.856
MB	*0.931*	−0.449	0.709	0.140	−0.280	−0.329	0.192	−0.120	0.831	0.738	0.472	*1*	−0.655
Pharynx	−0.841	−0.205	−0.679	−0.359	0.103	0.811	−0.197	0.347	−0.614	−0.261	−0.856	−0.655	*1*

Note: Values in italic are different from 0 with a significance level α = 0.05.

#### PCA and hierarchical clustering of P. hippeastri

**Figure 7:**
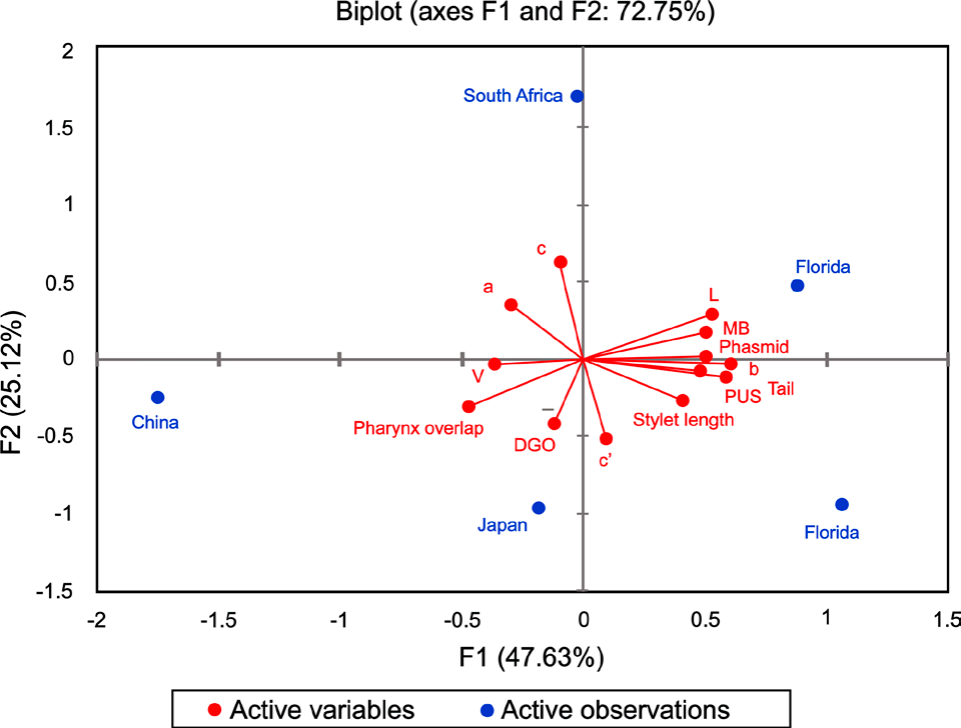
PCA analysis of the different population of *P. hippeastri*.

The principal component analysis was performed to study the variation within the populations of *P. hippeastri* (Fig. 7). An accumulated variability of 72.75% was detected in the female by the F1 (47.63%) and F2 (25.12%). All characters exhibited positive correlations among the populations and were responsible for the variability of the F1, except for *a*, *c*, V, DGO, and pharynx (Fig. 7). Some characters such as body length, *b*, MB, and tail length with 11.7, 13.2, 12.5, and 11 percent showed the most contribution to the variability and had a positive and high correlation with the F1.

**Figure 8:**
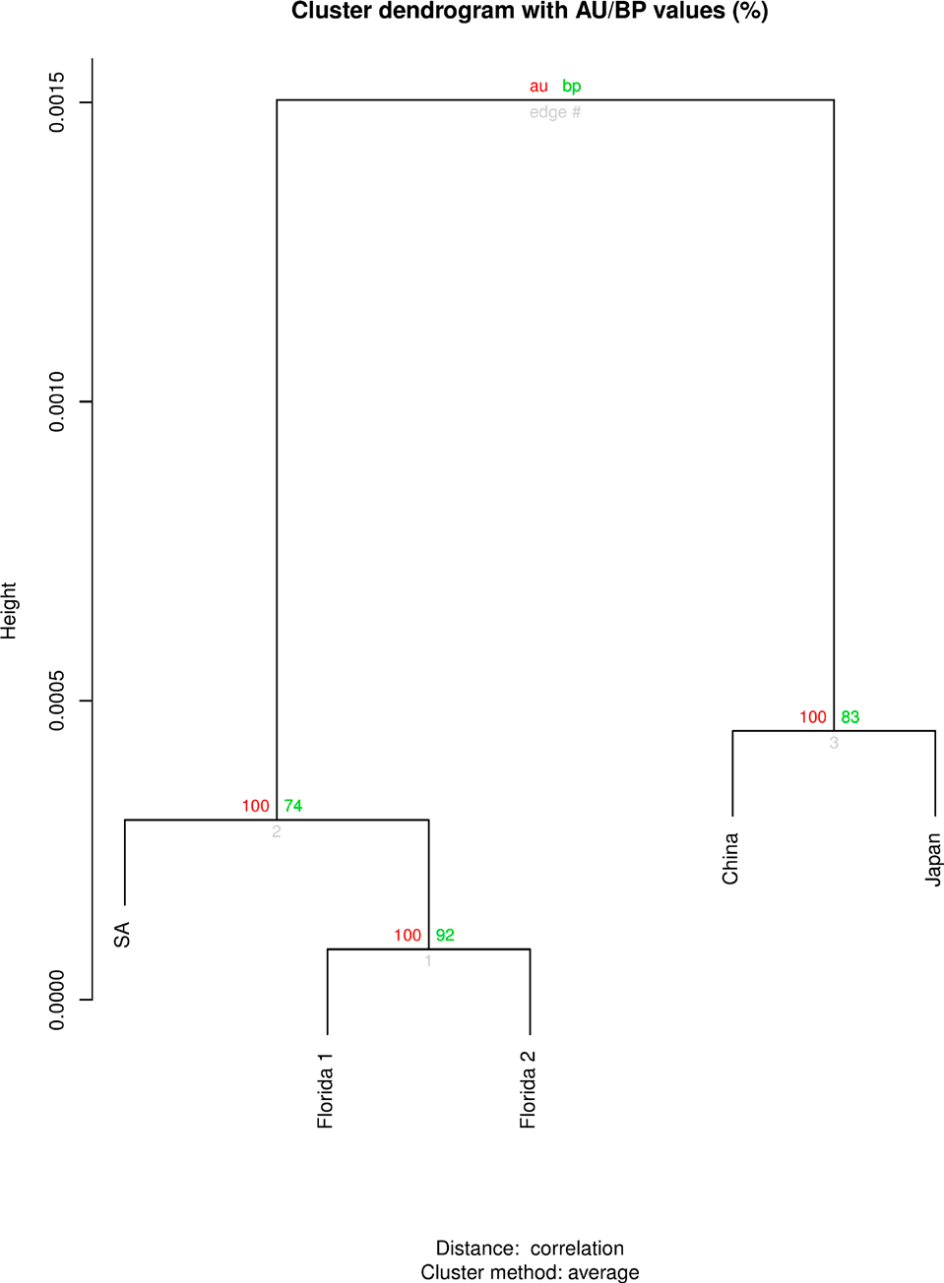
Cluster dendrogram for different populations of *P. hippeastri* using morphometric data. Red values represent AU (approximated unbiased) values. Green values on the right branch indicate BP (bootstrap probability). Florida 1 (Inserra et al. 2007) and Florida 2 (De Luca et al. 2010).

The dendrogram (Fig. 8) showed the average linkage method of hierarchical clustering with *p*-values of five populations of *P. hippeastri* from different localities in the world (Inserra et al., 2007; De Luca et al., 2010; Gu et al., 2014; Wang et al., 2016; present study). The hierarchical analysis based on important morphometric characters clustered the *P. hippeastri* populations studied into two groups. One group consisted of populations from the USA (Florida) and South Africa with 100 (AU: approximately unbiased) values and the second group consisted of populations from China and Japan with 100AU values (Fig. 8). This analysis demonstrated that the South African populations are very closely related to American populations. Moreover, the geographic pattern comprises of three continents with two of them (Africa and America) plotting completely separate from the Asian populations of *P. hippeastri*.

#### DNA characterization

The 18S rDNA (859 bp), ITS rDNA (910 bp), 28S rRNA (707 and 713 bp), and COI (mtDNA) (382 bp) gene fragments of the *P. hippeastri* studied during this research were amplified and compared using Nblast. The 18S rDNA of our population (MH324470) is similar to those of populations from the Israel and China (KJ001716 and KY424116; 99% identity) with three and five nucleotide difference, respectively. In addition, it is similar to a Dutch population of *P. scribneri* (EU669958; 99% identity) with two nucleotide differences. The ITS rDNA of the population studied (MH324471) is similar to those of populations from the USA (FN554886 and FN554888; 99% identity with one nucleotide difference only). Compared to the Chinese population of the same species, the Potchefstroom population showed two nucleotide differences (KY424236; KY424237; 99% identity). The 28S rDNA of the population studied (MH324472; MH324473) are similar with populations from the USA (FN554879 and FM994117; 99% identity) and China (KP161608 and KC796707; 99% identity), with one and two nucleotide difference, respectively. The COI gene of mtDNA of the population studied (MH324474) is similar to those of populations from China (KY424098 and KY424099; 97% identity), with 11 and 10 nucleotide differences, respectively.

#### Phylogenetic analysis

**Figure 9:**
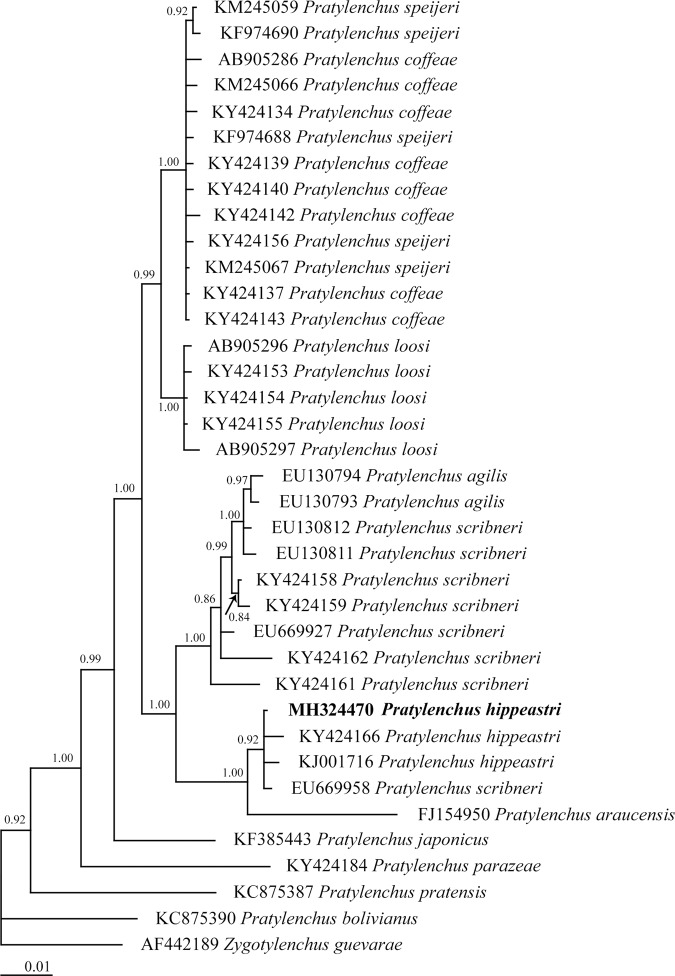
The Bayesian inference tree of *Pratylenchus hippeastri* from South Africa and other related taxa based on the sequences from 18S rDNA under GTR+I+G model (−*lnL* = 5,036.0855; AIC = 10,236.171; freqA = 0.2586; freqC = 0.2234; freqG = 0.2663; freqT = 0.2517; R(a) [AC] = 1.29106; R(b) [AG] = 2.99041; R(c) [AT] = 1.68788; R(d) [CG] = 0.89263; R(e) [CT] = 6.4881; R(f) [GT] = 1; p-inv = 0.5010; Shape = 0.4870).

**Figure 10:**
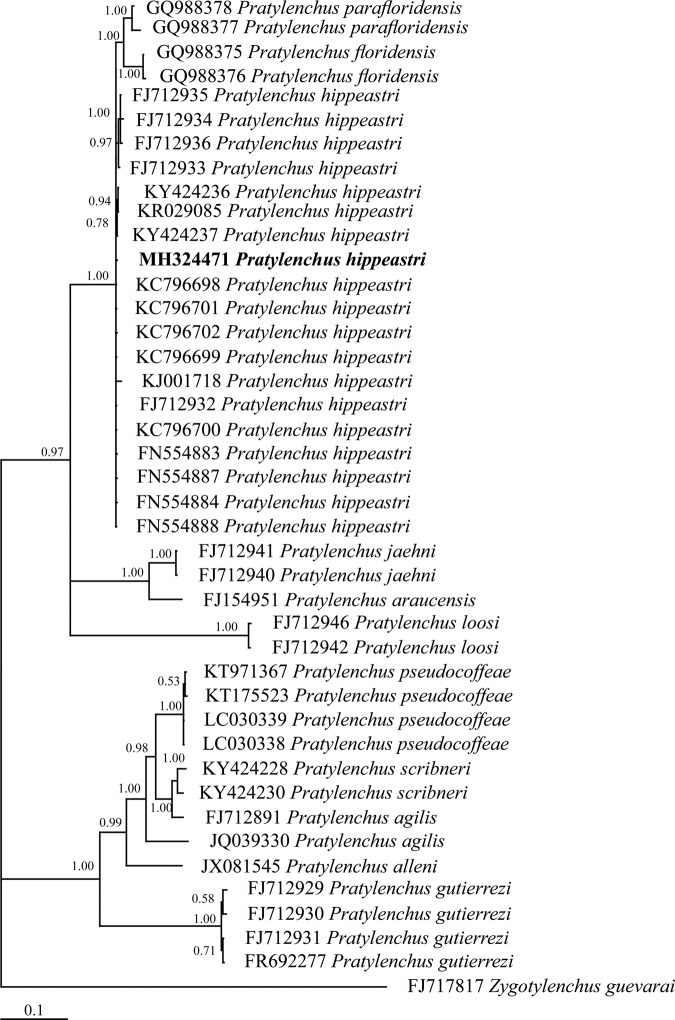
The Bayesian inference tree of *Pratylenchus hippeastri* from South Africa and other related taxa based on the sequences from ITS rDNA under GTR+I+G model (−*lnL* = 7,745.2851; AIC = 15,674.5702; freqA = 0.2437; freqC = 0.2123; freqG = 0.255; freqT = 0.2889; R(a) [AC] = 1.07478; R(b) [AG] = 2.56737; R(c) [AT] = 1.63147; R(d) [CG] = 0.53909; R(e) [CT] = 2.91622; R(f) [GT] = 1; p-inv = 0.2300; Shape = 1.3540).

**Figure 11:**
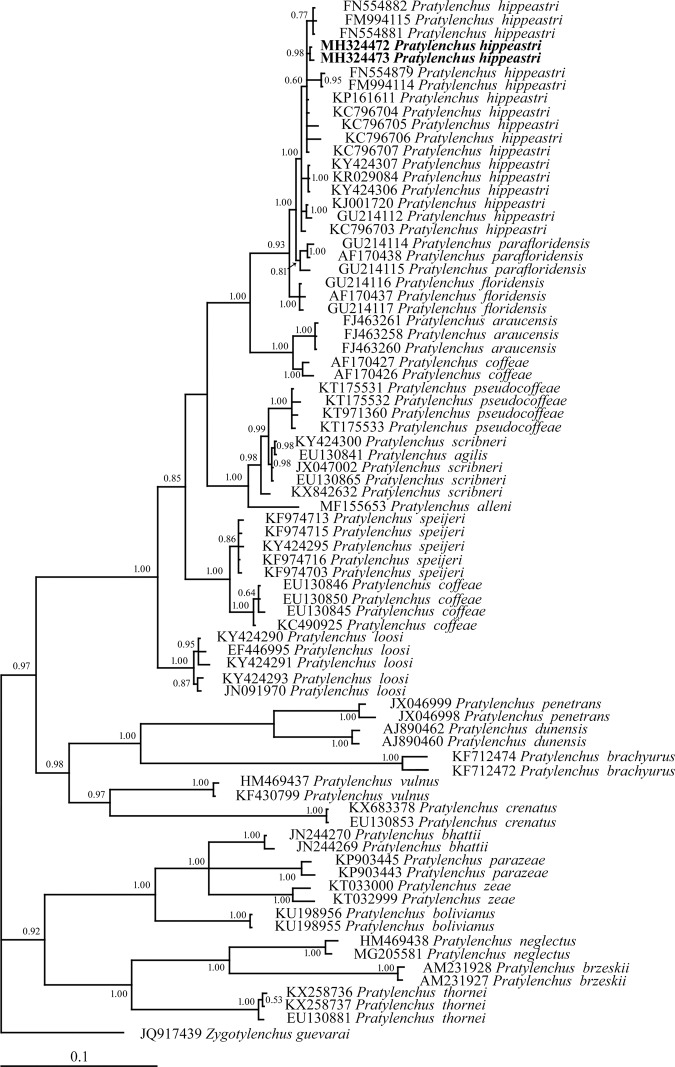
The Bayesian inference tree of *Pratylenchus hippeastri* from South Africa and other related taxa based on the sequences from 28S rDNA under GTR+I+G model (−*lnL* = 7,451.6325; AIC = 15,235.265; freqA = 0.2081; freqC = 0.2296; freqG = 0.3327; freqT = 0.2296; R(a) [AC] = 0.83418; R(b) [AG] = 2.50021; R(c) [AT] = 1.25212; R(d) [CG] = 0.34218; R(e) [CT] = 4.6954; R(f) [GT] = 1; p-inv = 0.2510; Shape = 0.6830).

**Figure 12:**
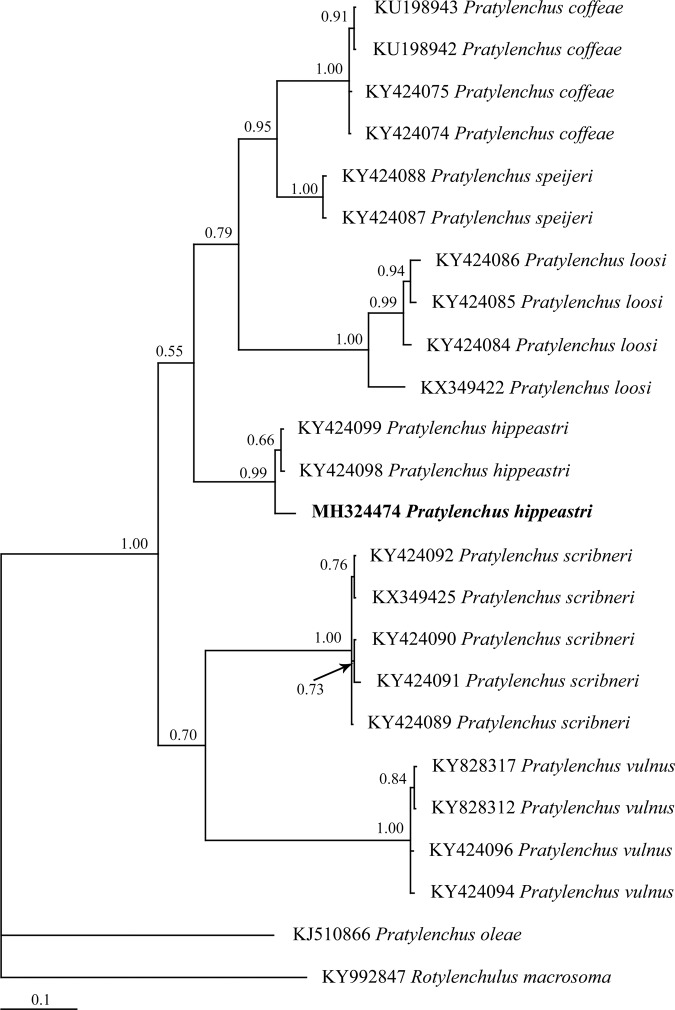
The Bayesian inference tree of *Pratylenchus hippeastri* from South Africa and other related taxa based on the sequences from COI of mtDNA under GTR+I+G model (−*lnL* = 2,667.1378; AIC = 5,446.2756; freqA = 0.2552; freqC = 0.0926; freqG = 0.1917; freqT = 0.4604; R(a) [AC] = 0.01; R(b) [AG] = 9.49586; R(c) [AT] = 2.67386; R(d) [CG] = 3.1383; R(e) [CT] = 8.02121; R(f) [GT] = 1; p-inv = 0.2150; Shape = 0.4730).

Regarding *P. hippeastri*, the Bayesian inference trees constructed on the basis of the 18S rDNA, ITS rDNA, D2 to D3 segment of 28S and COI of mtDNA sequences are shown in Figures 9 to 12, respectively. All populations of *P. hippeastri* are placed in one group with highly supported bootstrap values of 100%. Based on the phylogenetic analysis using ITS (Fig. 10) and 28S rDNA, *P. hippeastri* placed close to *P. floridensis* (De Luca et al., 2010) and *P. parafloridensis* (De Luca et al., 2010).

According to the 28S rDNA phylogenetic tree results (Fig. 11), the South African populations are more closely related to the Floridian (USA) (FM994114; FN554879) populations than to the Chinese populations (KC796706; KC796707). However, based on 18S rDNA (Fig. 9), the South African population of *P. hippeastri* placed close to the same species from China (KY424166; KJ001716) as well as *P. scribneri* Steiner *in* Sherbakoff and Stanley (1943) from the Netherlands (EU669958), and *P. araucensis* (Múnera et al., 2009) from Germany (FJ154950). The COI phylogenetic results (Fig. 12) indicated that the South African population grouped with the Chinese population of *P. hippeastri* (KY424098; KY424099).

## Discussion

The genus *Hirschmanniella* comprises 24 nominal species according to Loof (1991) and 29 nominal species are considered by Khun et al. (2015), however, the identification of the species only based on the morphometric characters is challengeable and needs molecular approaches to assist in the precise diagnose (Khun et al., 2015). The result obtained in this study is in agreement with those obtained by Van den Berg et al. (2009) and Khun et al. (2015). Albeit, the morphological variations have been indicated by Khun et al. (2015) due to intraspecific variation. *Hirschmanniella mucronata* was a sister taxon to *H. kwazuna* and *H. loofi*, with strong support as stated by Khun et al. (2015). *Hirschmanniella oryzae* and *H. belli* also form a group, which is stated by Khun et al. (2015). However, they differ in body length (1.61-2.22 mm in female and 1.4-1.90 mm in male for *H. belli* vs 1.03-1.63 mm in female and 1.01-1.40 mm in male for *H. oryzae*), stylet length (20-22 µm for *H. belli* vs 15-19 µm for *H. oryzae*), and spicule length (31-36 µm for *H. belli* vs 18-26 µm for *H. oryzae*) (Sher, 1968). The phylogenetic result also placed *H. anchoryzae* together with *H. pomponiensis*, *H. santarosae*, and *H. halophila*. However, it differs with *H. santarosae* in lower value of the body length (1.7 mm vs 1.3 mm) and male (present vs absent). In comparison with *H. pomponiensis*, they differ in female body length (1.7-1.9 mm vs 1.7–2.4 mm), tail length (96–106 µm in female and 81–87 µm in male vs 99–189 µm in female and 97–133 µm in male), spicule length (27–30 µm vs 32–40 µm) and gubernaculum length (8.6–8.7 µm vs 10–13 µm). In comparison with *H. halophila*, they differ in lower value of the body (1740 µm vs 1260 µm) and mucro at tail tip (1 *vs* 2-4). The placement of the species based on 28S rDNA is in agreement by the PCA analysis which *H. anchoryzae* and *H. halophila* place close each other.

The usefulness of morphometric analyses, due to significant correlations between some morphometric characteristics of females of the five populations of *P. hippeastri*, is suitable to find variation among populations. In addition, morphometric cluster analysis is also suggested that the variation among the *P. hippeastri* specimens as the South African and the USA populations were placed together, while the Chinese and Japanese populations group together, both with 100 bootstrap values.

Regarding morphometric analysis, the correlations of such measurements or indices have been studied by Divsalar et al. (2018) in the populations of *P. thornei.* Ryss (2002) used morphological traits to study the evolution among the species of *Pratylenchus*. However, the mentioned author did not report any correlation that possibly exists in the genus *Pratylenchus*. In our study, the body length showed correlation with the post uterine sac and tail, as well as the indices such as *b′*, which agreed with those reported by Divsalar et al. (2018) among the populations of *P. thornei*. Furthermore, Nguyen (2010) indicated that the canonical discriminant analysis grouped 10 populations of *P. coffeae* from Vietnam by using five morphometric characters of the male specimens successfully, representing of the usefulness of morphometric analysis among the specific species of *Pratylenchus*. Concerning to other plant-parasitic nematodes, Fortuner (1984) noted that the indices *a*, *c*, and *c’* are often useful to accurately identify species of *Helicotylenchus* (Steiner, 1945). Fortuner (1990) also revealed that the features related to body size (length and indices of *a* and *b*) showed a high correlation to each other for *Hirschmanniella belli*. Subbotin et al. (1999) also stated that morphometric character analysis is suitable for separating populations within the *H. avenae* group. As a result of this study, cluster analysis, according to morphometric characteristics, grouped the South African population of *P. hippeastri* close to the American populations, but in a separate group than the Asian populations.

The phylogenetic position of the *P. hippeastri* was studied by the use of rDNA (such as 18S rDNA, ITS rDNA, and 28S rDNA) and mtDNA (COI gene) sequences of nematodes belonging to the genus *Pratylenchus* from GenBank. The consensus trees based on ITS and 28S rDNA showed that the *P. hippeastri* is represented by a monophyletic group. Inserra et al. (2007) indicated that *P. hippeastri* is close to *P. scribneri*, which is not shown by the phylogenetic analysis. These two species, despite the differences mentioned by Inserra et al. (2007), the SEM observations indicated that they differ in the lateral field (three incisures reach the end of the female tail in *P. hippeastri* vs four incisures reach the end of the female tail in *P. scribneri*) (see Castillo and Vovlas, 2007). Except for the ITS rDNA, 28S rDNA, and COI of mtDNA, the 18S rDNA revealed that some populations of both species, possibly similar to each other, needed to be investigated deeply by morphology. Since the morphology of the molecularly identified *P. scribneri* (EU669958) is not accessible, judging on the similarity among that and with the two *P. hippeastri* (KJ001716; KY424166) is not logic. In addition, based on the phylogenetic analysis, *P. hippeastri* place close to *P. floridensis* and *P. parafloridensis* in one clade with 100 posterior probability values, as indicated by De Luca et al. (2010) and Araya et al. (2016). These authors considered all three species as a complex under the name of ‘*hippeastri*’. The mentioned amphimictic species recovered from the same geographical region (Florida, USA) differ in spermatheca (non-functional despite the male existence in *P. hippeastri* vs functional in *P. floridensis* and *P. parafloridensis*) and tail end morphology (almost indented in *P. hippeastri* vs almost smooth in *P. floridensis* and *P. parafloridensis*) (De Luca et al., 2010). The pairwise genetic distance of these three species revealed that *P. hippeastri* has 0.016 and 0.012 difference with *P. floridensis* and *P. parafloridensis*, respectively. However, *P. floridensis* showed 0.018 differences with *P. parafloridensis*. Despite all differences, more specimens need to be investigated by SEM morphology to understand that are them complex, putative, or separate species?
